# Vaginal microbiome and serum metabolite differences in late gestation commercial sows at risk for pelvic organ prolapse

**DOI:** 10.1038/s41598-021-85367-3

**Published:** 2021-03-17

**Authors:** Zoë E. Kiefer, Lucas R. Koester, Lucas Showman, Jamie M. Studer, Amanda L. Chipman, Aileen F. Keating, Stephan Schmitz-Esser, Jason W. Ross

**Affiliations:** 1 Department of Animal Science, Iowa State University, Iowa State University, 2356 Kildee Hall, Ames, IA 50011 USA; 2 Department of Veterinary Microbiology and Preventive Medicine, Iowa State University, Ames, IA USA; 3 Interdepartmental Microbiology Graduate Program, Iowa State University, Ames, IA USA; 4 W. M. Keck Metabolomics Research Laboratory, Iowa State University, Ames, IA USA; 5 Iowa Pork Industry Center, Ames, IA USA

**Keywords:** Microbiology, Molecular biology, Physiology

## Abstract

Sow mortality attributable to pelvic organ prolapse (POP) has increased in the U.S. swine industry and continues to worsen. Two main objectives of this study were, (1) to develop a perineal scoring system that can be correlated with POP risk, and (2) identify POP risk-associated biological factors. To assess POP risk during late gestation, sows (n = 213) were scored using a newly developed perineal scoring (PS) system. Sows scored as PS1 (low), PS2 (moderate), or PS3 (high) based on POP risk. Subsequently, 1.5, 0.8, and 23.1% of sows scored PS1, PS2, or PS3, respectively, experienced POP. To identify biomarkers, serum and vaginal swabs were collected from late gestation sows differing in PS. Using GC–MS, 82 serum metabolite differences between PS1 and PS3 animals (*P* < 0.05) were identified. Vaginal swabs were utilized for 16S rRNA gene sequencing and differences in vaginal microbiomes between PS1 and PS3 animals were detected on a community level (*P* < 0.01) along with differences in abundances of 89 operational taxonomic units (*P* < 0.05). Collectively, these data demonstrate that sows with greater POP risk have differential serum metabolites and vaginal microflora. Additionally, an initial and novel characterization of the sow vaginal microbiome was determined.

## Introduction

Sow reproductive performance across the U.S. swine industry has increased in the past decade culminating in some farms achieving thirty pigs weaned per sow per year. Despite marked improvements in sow key performance indicators (farrowing rate, litter size, pigs weaned), there has also been a substantial increase in sow mortality due to pelvic organ prolapse (POP) during late gestation and early lactation^1^. In a survey of the U.S. swine industry POP was determined to contribute to approximately 21% of sow deaths annually. Pelvic organ prolapse is an anatomical disorder characterized by one or more pelvic organs (uterus, rectum and/or vagina) pressing up against or out of the vagina^2^. In sows, POP occurs predominantly within a few days of farrowing and can result in loss of both the sow and her offspring. The incidence of sow POP continues to increase, yet there is a lack of mitigation strategies since the biological causative underpinnings are ill-defined^1^.

The surfaces and cavities of all mammalian species exposed to the environment are host to microorganisms^3^ and can have substantial influence on animal health and well-being. Recent discoveries in swine have revealed the contribution of the microbiota to gut function^4,5^. Information on the vaginal microbiome in swine is limited and, in some cases, focused on genetic lines used in commercial production^6–9^. Findings in other species support the notion that changes in the microbiota of the reproductive tract can compromise reproductive function; as alterations to and increased diversity in the vaginal microbiome affect susceptibility to gynecologic infections^10^.

Serum contains biomarkers including lipids, amino acids, peptides, nucleic acids, organic acids, vitamins, thiols and carbohydrates. These are important in biological systems and have the ability to assist further understanding of disease phenotypes^11^. Non-targeted metabolomics is a global unbiased analysis of small-molecule metabolites present in a given biological sample^12^. The objective of this study was to develop a perineal scoring system to evaluate risk of POP of sows during late gestation and additionally assess differences in vaginal microbial populations and molecular features associated with POP risk to serve as potential biomarkers to better understand biological alterations associated with POP. The hypothesis that both the vaginal microbiome and serum small-molecule repertoires would differ between sows with differing risk of POP was tested in late gestation sows.

## Results

### Differences in perineal score is associated with differing risk of pelvic organ prolapse

**Figure 1:**
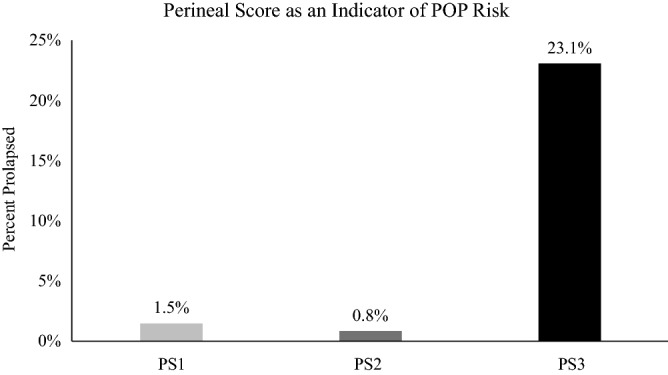
Perineal score in late gestation as an indicator of pelvic organ prolapse (POP) risk. Perineal scoring was conducted on sows (n = 213) during late gestation (days 105–117). Sows assigned a PS1 (n = 68), PS2 (n = 119) and PS3 (n = 26) were assumed low, medium or high risk, respectively, for POP. As predicted, 23.1% of PS3 scored sows subsequently died due to POP while 1.5 and 0.8% of sows scoring PS1 and PS2, died respectively. These data demonstrate the ability to distinguish differential risk of POP for animals during late gestation.

Of the 213 sows assigned a perineal score (PS) during late gestation, 68, 119 and 26 were assigned PS1, PS2 and PS3, respectively. Retrospectively, there was no difference (*P* = 0.81) in the number of sows that experience POP between PS1 (1.5%) and PS2 (0.8%) while sows that scored a PS3 (23.1%) had greater (*P* < 0.01) incidence of POP compared to PS1 or PS2 scored sows (Fig. 1). Additionally, parity had no effect on PS (*P* = 0.54).

### 16S rRNA gene amplicon sequencing identified association between vaginal microbiota and perineal score

**Figure 2:**
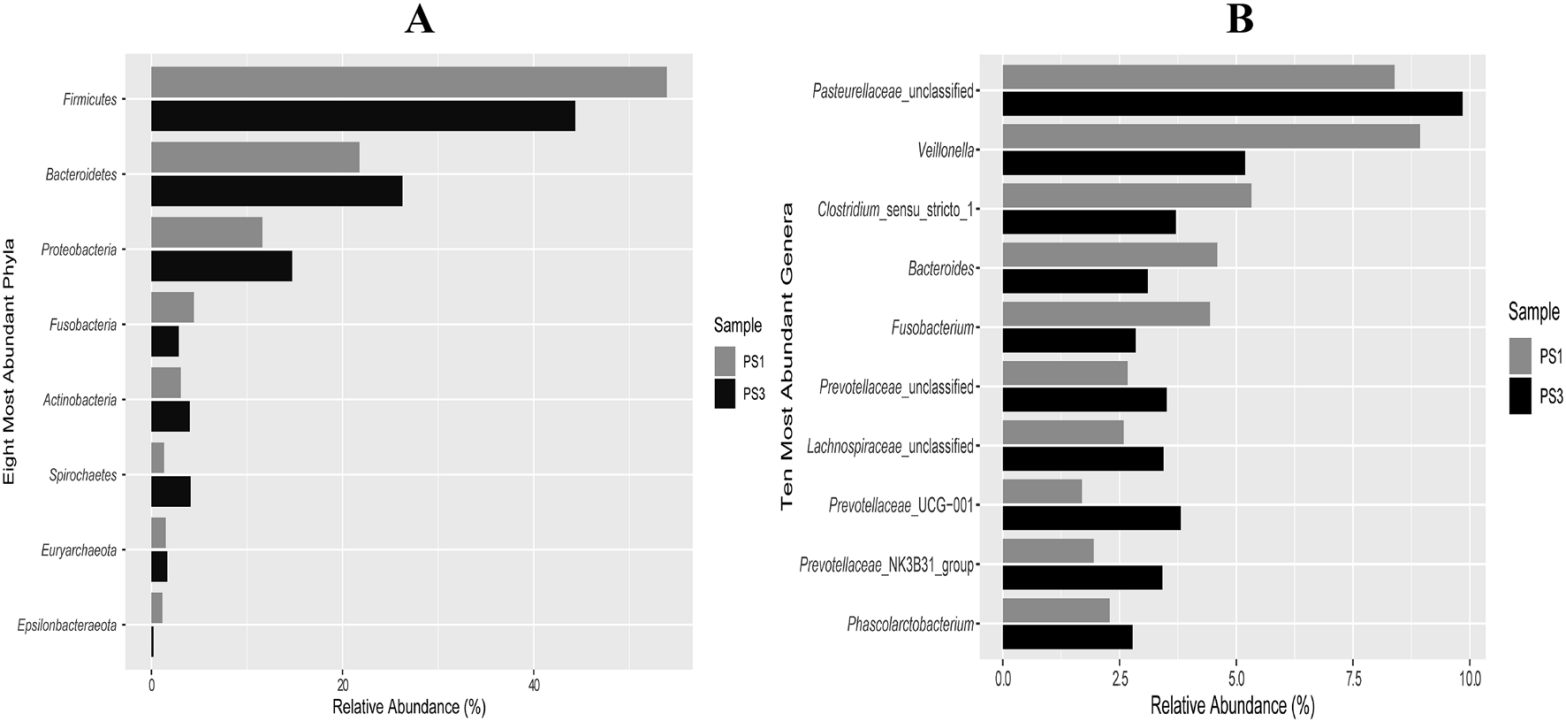
Most abundant microbial phyla (**A**) and genera (**B**) in vaginal swabs from sows with low and high risk for pelivc organ prolapse (POP). (**A**) Vaginal swabs were collected from sows with low (PS1) and high (PS3) risk for POP and microbial detection and abundance were determined through 16 s rRNA sequencing and analysis. Spirochaetes abundance between PS1 and PS3 sows was significantly different (*P* < 0.05). (**B**) Stacked bar charts comparing the ten most abundant vaginal microbial genera. Relative abundance of vaginal swab microbial communities on a genus level represent the mean across each perineal score. Animals assumed low risk for POP are considered PS1 and high risk animals for POP are considered PS3.

Of 6437 operational taxonomic units (OTUs) obtained from 42 samples (23 PS1 and 19 PS3), A total of 1711 OTUs remained after quality control and removal of OTUs representing less than ten sequences. The average number of sequences per sample was 13,353 with a standard deviation of 8422. The majority of reads were bacterial (98.27%), and 1.73% were archaeal. Alpha diversity estimators revealed no significant differences between samples regarding species richness, community evenness and diversity (Supplementary Fig. S1). Out of the 1711 OTUs 26 phyla were identified. *Firmicutes, Bacteroidetes, Proteobacteria, Actinobacteria,* and *Fusobacteria* were the most abundant, representing 49.6%, 24.0%, 12.6%, 4.0%, and 3.3% of all reads, respectively (Fig. 2A). There were 571 genera identified of which the most abundant included *Pasteurellaceae* unclassified (7.6%)*, Veillonella* (6.2%)*, Clostridium* cluster I (4.5%), *Bacteroides* (3.4%), and *Prevotellaceae* unclassified (3.3%) (Fig. 2B). The 50 most abundant vaginal tract OTUs are reported in Supplementary Table S1. The most abundant OTU (OTU 1) was identified as *Veillonella*, and accounted for 6.2% of all the reads. OTUs 2, 3, 4, and 5 were classified as *Pasteurellaceae* unclassified, *Fusobacterium*, *Prevotellaceae UCG-001*, and *Phascolarctobacterium*, respectively, and accounted for 5.2%, 2.6%, 2.4%, 2.2%, and 2.1%, respectively.

**Figure 3:**
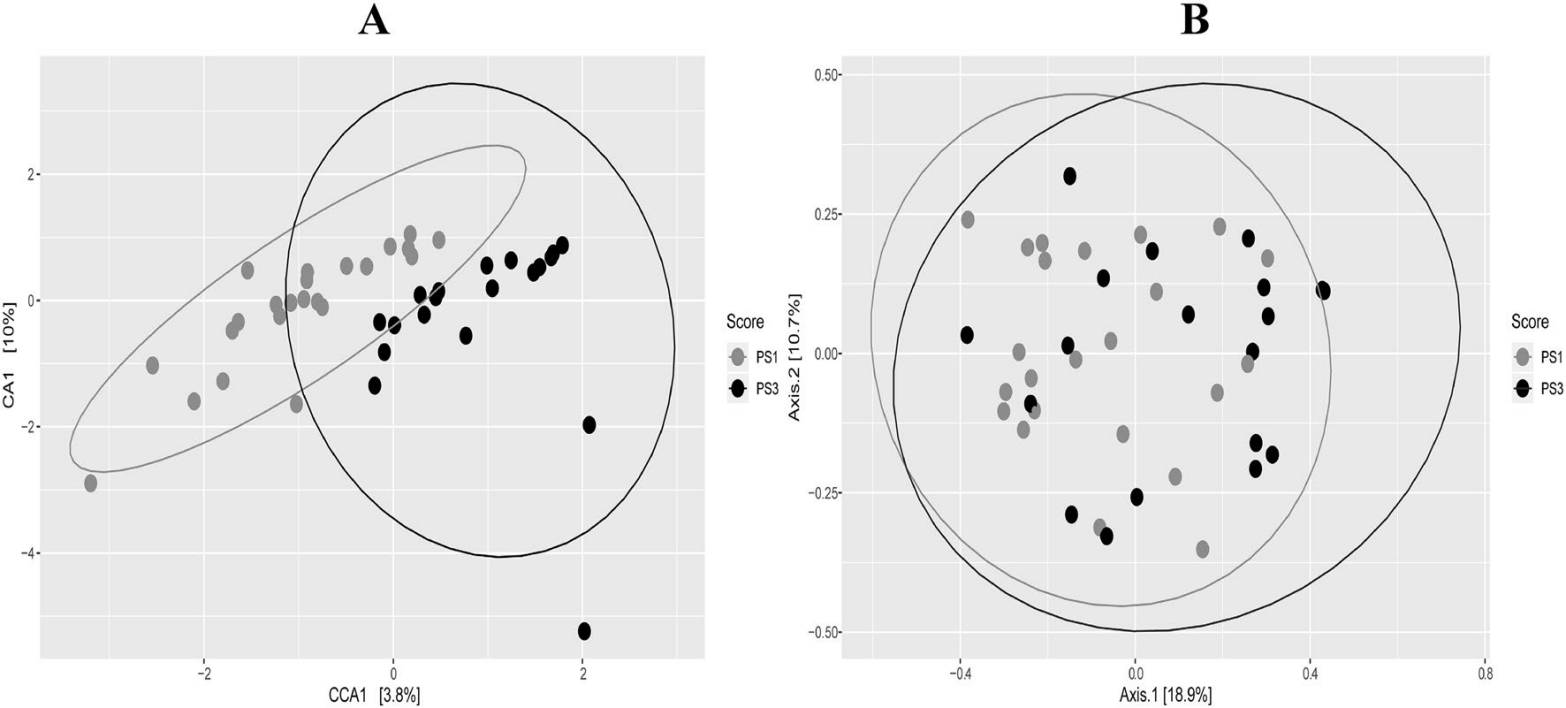
Vaginal swab community comparisons. (**A**) Canonical correspondence analysis showing maximum variation between samples in community due to perineal score (PS). Data for low risk animals (PS1) are depicted in light grey and high-risk animals (PS3) are in black. All points represent Bray Curtis dissimilarity measures for each sample. (**B**) Principal Coordinates Analysis showing differences in beta diversity of vaginal microbial communities from sows with different perineal scores (PS). All points represent Bray Curtis dissimilarity measures for each sample. Ellipses were based on a multivariate normal distribution of data points. There were statistical differences in overall microbial communities found using ANOSIM due to PS (*P* < 0.01).

**Figure 4:**
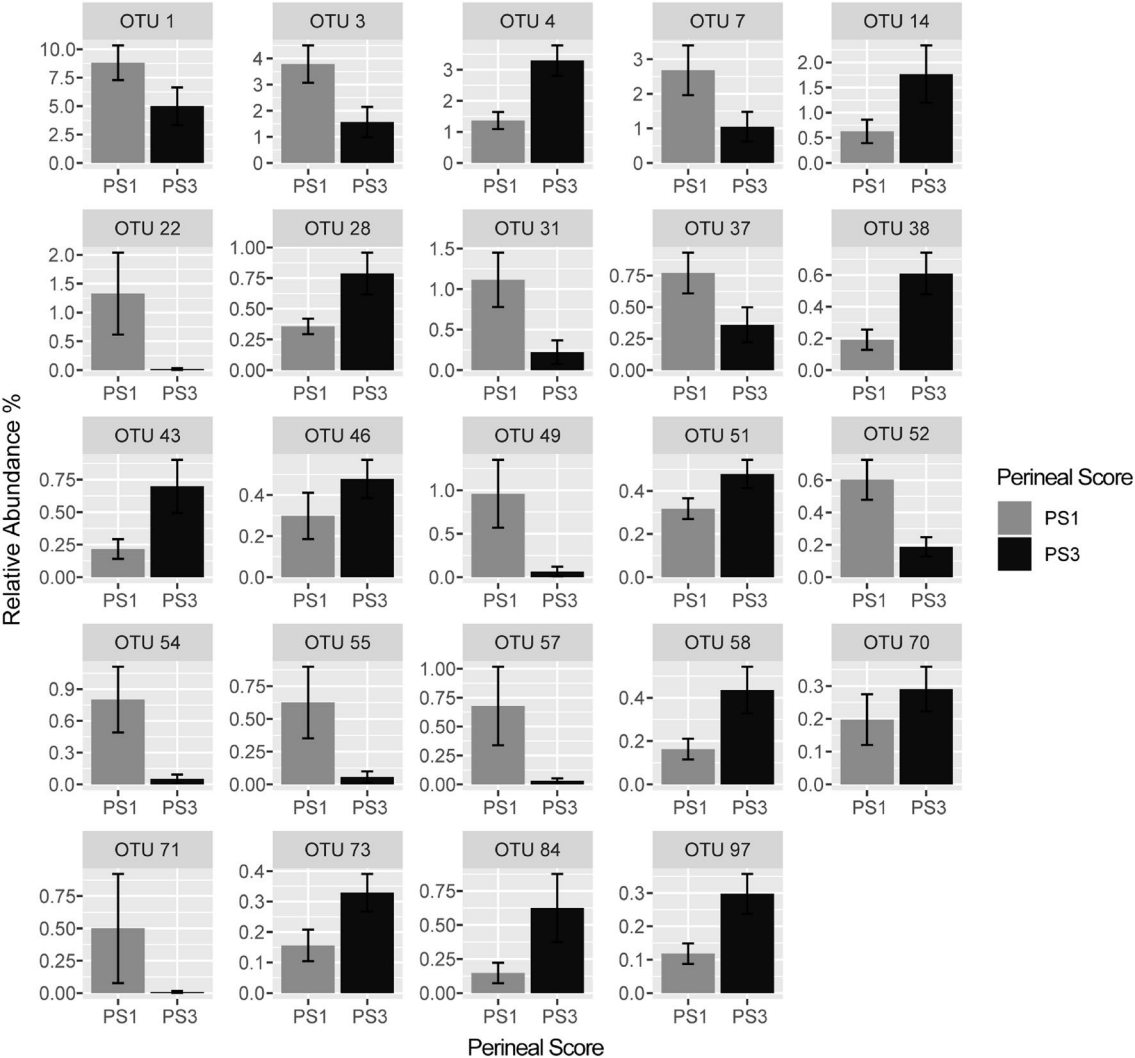
Different OTUs in the vaginal microbiota of sows with different perineal scores (PS). Significantly different (*P* < 0.05) OTUs were identified with LEfSe. Only significant OTUs within the 100 most abundant are shown. Error bars represent the standard error of the mean. Out of all significantly different OTUs, 24 were considered significantly different among the 100 most abundant OTUs. Out of these 24 OTUs, 12 were more abundant in PS1 animals and 12 were more abundant in PS3 animals. See Table 1 for more details.

When comparing the microbiota of PS1 and PS3 sows, there were differences on a whole community level using analysis of similarity (ANOSIM) (*P* < 0.01) and some separation using canonical correspondence analysis (Fig. 3A). However, principal coordinate analyses revealed no distinct clustering of PS1 and PS3 samples (Fig. 3B). Using Linear Discriminant Analysis (LDA) Effect Size (LEfSe), abundance differences (*P* < 0.05) in 89 total OTUs were observed. Of these, 26 OTUs were more abundant in PS1 and 68 were more abundant in PS3. Out of the 89 significantly different OTUs, 24 were within the 100 most abundant OTUs (Fig. 4, Table 1). Of those, 12 OTUs were more abundant in PS1, including OTUs 1, 3, 7, 22, 31, 37, 49, 52, 54, 55, 57, and 71. There were also 12 OTUs that were more abundant in PS3, including OTUs 4, 14, 28, 38, 43, 46, 51, 58, 70, 73, 84, and 97. A higher abundance of *Prevotellaceae,* and *Treponema* within several OTUs of each were noted in PS3 animals compared to PS1. In addition, a *Streptococcus dysgalactiae* OTU was more abundant (*P* < 0.01) in PS3 sows.

**Table 1:** Differences in OTUs between vaginal microbiomes of PS1 and PS3 sows.

OTU	Taxonomy (Silva v132)	NCBI BLAST Classification	LDA^1^	PS^2^	*P-*value
OTU 1	*Veillonella*	*Veillonella caviae* PV1	4.08	1	0.03
OTU 3	*Fusobacterium*	*Fusobacterium gastrosuis CDW1*	4.13	1	< 0.01
OTU 4	*Prevotellaceae UCG-001*	*Duncaniella sp. TLL-A3*	4.08	3	< 0.01
OTU 7	*Parvimonas*	*Parvimonas sp. KA00067*	3.70	1	0.02
OTU 14	*Bacteroidales unclassified*	*Muribaculum sp. S4*	3.82	3	0.04
OTU 22	*Porphyromonas*	*Porphyromonas somerae KA00683*	3.27	1	< 0.01
OTU 28	*Prevotellaceae NK3B31 group*	*Prevotellaceae bacterium*	3.63	3	0.03
OTU 31	*Anaerococcus*	*Anaerococcus tetradius*	3.74	1	< 0.01
OTU 37	*Streptococcus*	*Streptococcus hyovaginalis TRG26*	2.84	1	0.04
OTU 38	*Treponema 2*	*Treponema bryantii,*	3.47	3	0.02
OTU 43	*Ruminococcus 1*	*Bacterium MA2007*	3.36	3	0.02
OTU 46	*Prevotellaceae UCG-001*	*Muribaculum sp. S4*	3.23	3	< 0.01
OTU 49	*Porphyromonas*	*Porphyromonas katsikii JF5581*	3.76	1	< 0.01
OTU 51	*Lachnospiraceae XPB1014 group*	*Absiella sp. strain 1XD42-72*	2.99	3	0.05
OTU 52	*Streptococcus*	*Streptococcus gallolyticus 96L8*	3.14	1	0.02
OTU 54	*Ezakiella*	*Clostridiales bacterium S9 PR-1*	3.58	1	< 0.01
OTU 55	*Gallicola*	*Peptoniphilaceae bacterium SIT14*	3.32	1	< 0.01
OTU 57	*Porphyromonas*	*Porphyromonadaceae bacterium FC4*	3.29	1	< 0.01
OTU 58	*Prevotellaceae unclassified*	*Prevotella copri DSM 108494*	3.45	3	0.03
OTU 70	*Treponema 2*	*Candidatus Treponema suis*	3.31	3	0.04
OTU 71	*Murdochiella*	*Levyella sp. Marseille-P3170*	2.20	1	< 0.01
OTU 73	*Treponema 2*	*Treponema porcinum 14V28*	3.27	3	0.01
OTU 84	*Streptococcus*	*Streptococcus dysgalactiae PK*	2.86	3	< 0.01
OTU 97	*Lachnospiraceae unclassified*	*Kineothrix alysoides KNHs209*	2.92	3	< 0.01

Individual microbes were assigned in ordered of abundance and classified into operational taxonomic units (OTUs).

^1^Linear discriminate analysis (LDA) was used for comparison between perineal scores.

^2^Sows were assigned a perineal score (PS) based on their relative risk of experiencing a pelvic organ prolapse (POP). Sows assigned PS1 were presumed low risk for POP while sows assigned PS3 were presumed high risk for POP.

### Serum small molecule metabolites differ with POP risk

**Figure 5:**
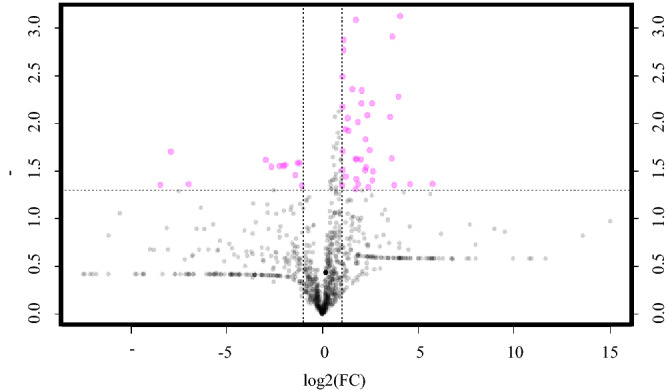
Serum metabolites in sows differing in perineal score (PS) and pelvic organ prolapse (POP) risk. This plot represents important features and demonstrates those which are different between sows with a PS3 (high risk for POP) compared to PS1 (low risk for POP). The pink circles represent features above the threshold (twofold change and *P* < 0.05). PS1 averages were set as the base level and PS3 values of individual metabolites are represented by points. The pink dots represent the individual metabolites that are considered significantly different between PS. Pink dots in the upper right are metabolites that are more abundant, on average, in PS3 animals, and pink dots in the upper left less abundant, on average, in PS3 animals. Note both fold changes and *P*-values are log transformed. The further the position from the (0,0), the greater level of statistical significance for that the feature.

**Figure 6:**
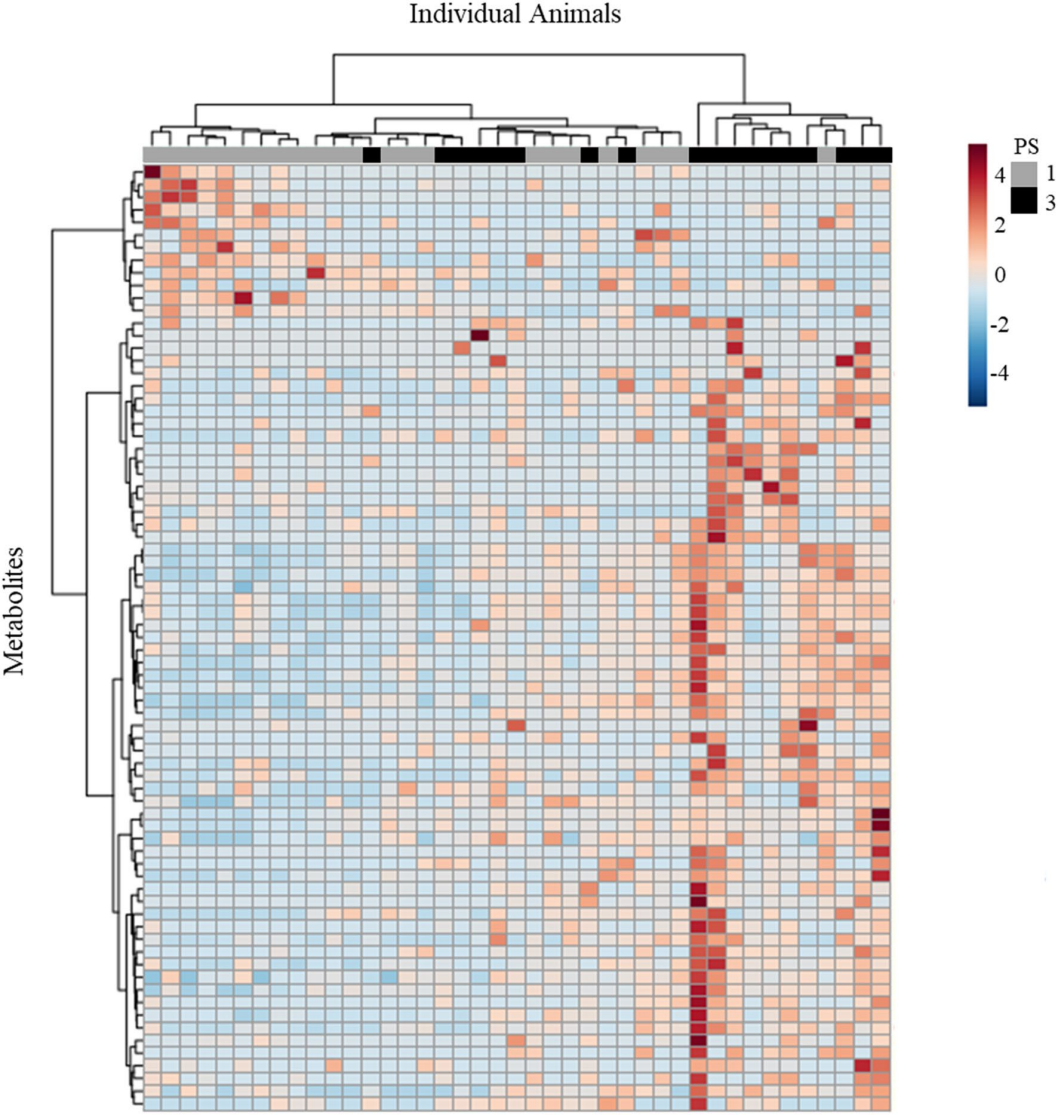
Differences in serum metabolites between PS1 and PS3 sows. This heat map displaying the 75 molecular features with the highest statistical significance (lowest p-values), illustrates relative abundance of serum metabolites (represented by color) between low POP risk (PS1) and high POP risk (PS3) sows. PS3 scored sows (black) cluster to the left of the dendrogram separate from PS1 scored sows (grey).

Following GC–MS, a total of 960 molecular features were detected in the 44 serum samples used (25 PS1 and 19 PS3). There were 82 differently abundant metabolites between treatment groups (*P* < 0.05), and four were more abundant in PS1 animals (Fig. 5). Overall metabolites between PS1 and PS3 animals were determined and an expected overlap was noted, but distinct differences were also observed (Supplementary Fig. S2). Grouping of PS based on serum metabolite abundances was also determined (Fig. 6). Out of the 960 molecular features detected, 93 of them were identifiable. When evaluating just the 93 identified metabolites 16 differed (*P* < 0.05) between PS1 and PS3 (Table 2). Metabolites such as L-methionine, L-alanine, 2-aminobutanoic acid, lactic acid, D-glucose and several others were more abundant (*P* < 0.05) in PS3 compared to PS1 scored sows. All 16 of the identified differential metabolites were more abundant in PS3 animals. One of particular interest is D-Fructose which was 5.3-fold more abundant in PS3 sows and had a 78.5% accuracy as a potential POP risk biomarker. There were several other potential biomarkers identified all above 74% area under the curve (Supplementary Table S2). Nine of these potential biomarkers met the biomarker criteria, however, eight are currently not identifiable.

**Table 2:** Small molecule metabolites in serum from sows with differing risk of POP.

	Perineal score^1^			
Metabolite^2^	PS1	PS3	Fold Change	*P*-value
d-Fructose	1.48E−11	5.80E−10	5.29	< 0.01
Pseudo uridine	3.93E−12	1.28E−10	5.02	< 0.01
l-Methionine	2.92E−11	7.92E−10	4.76	0.01
l-Alanine	2.51E−10	7.36E−09	4.87	0.02
2-Aminobutanoic acid	2.30E−12	8.18E−11	5.16	0.02
Ethanolamine	2.98E−12	1.04E−10	5.12	0.02
l-Glutamic acid	1.49E−11	5.03E−10	5.07	0.03
l-Proline	1.80E−10	5.12E−09	4.83	0.04
Glycine	3.19E−10	9.25E−09	4.86	0.04
l-Tryptophan	2.46E−11	6.53E−10	4.73	0.04
Myo-Inositol	2.47E−10	6.19E−09	4.65	0.04
Lactic acid	1.75E−09	4.17E−08	4.57	0.04
Hippuric acid	9.31E−12	7.94E−11	3.09	0.04
Oleic acid	7.71E−11	2.25E−09	4.87	0.05
d-Glucose	7.94E−10	1.97E−08	4.64	0.05
l-Asparagine	8.45E−12	2.47E−10	4.87	0.05

^1^Sows were assigned a perineal score based on their relative risk of experiencing a pelvic organ prolapse (POP). Sows assigned PS1 were presumed low risk for POP while sows assigned PS3 were presumed high risk for POP.

^2^Metabolite list only includes those that were identifiable and statistically significant (*P* < 0.05) between perineal scores. Values reported as moles per mg.

## Discussion

Despite dramatic improvements in reproductive performance in the U.S. swine industry, POP still remains an issue. Of all causes of sow mortality, 21% is due to POP, although the biological explanation remains unknown. This is considerably higher than other livestock species, such as the bovine, which experiences less than 1%^13^. Critical for the development of mitigation strategies is the better understanding of the biological events that precede POP. To this end, this study tested the hypothesis that a PS system could have utility in defining POP risk and further, that differential vaginal microbiota and serum metabolites would be associated with altered POP risk in sows. Our data demonstrates that sows with variable risk for POP can be identified during late gestation through phenotypic evaluation of the perineal region. This evaluation system of the perineal region of sows is significant for the swine industry as identification of animals with elevated risk for POP has proven difficult. While the PS was effective in assessing POP risk, the biological factors contributing to the phenotypic variation observed remain unknown.

Previous studies investigating the sow microbiome have not been conducted in commercial settings and/or were not conducted with pregnant sows, which could influence the environmental microbiota presence and potentially affect the vaginal microbiome^6–8^. Other studies have attempted to evaluate the vaginal microbiota in swine via in vitro culture^14^. While providing some novel information, cultivation approaches may not accurately capture the in vivo representation of the microbiota in swine as not all microorganisms grow effectively in a culture system. This study is our initial characterization of the vaginal microbiome of commercial sows during gestation. The data demonstrates a diverse microbial community within the vagina of commercial sows while also identifying differences in the vaginal microbiome between sows with differing risk for POP.

When comparing overall microbial community structure between sows with low and high risk of POP there were differences on a whole community level. It is well established that the most abundant microbe in the human vaginal microbiome is *Lactobacillus*^15^. In this study, *Lactobacillus* presence was observed, but at a much lower average abundance compared to human vaginal microbiomes. In agreement with this, other published pig vaginal microbiota studies also determined a relatively low overall abundance of *Lactobacillus*^6–9^. A higher abundance of *Streptococcus dysgalactiae* was noted in PS3 sows. *Streptococcus dysgalactiae* is a major pathogen in humans and animals and has also been associated with equine reproductive system infections^16,17^. Further, similarities in the genera of species present were consistent with previous studies on the swine vaginal microbiome. Sanglard et al.^8^ reported *Fusobacterium* presence in non-pregnant gilts at a relative abundance of 11.4%. In the current study *Fusobacterium* was observed in late gestation sows at a lower relative abundance (2.6%) and was greater in sows with low risk for POP compared to sows with high risk. A higher abundance of *Fusobacterium* in the sow vaginal microbiota with endometritis has been determined^6^. The most abundant microbe present in the vaginal microbiome of sows in this study was *Veillonella*, with a relative abundance of 6.2% which also differed in relative abundance between sows of different POP risk with a higher abundance of *Veillonella* in PS1 sows. Previous studies have reported that *Veillonella* is present in the vaginal microbiome of unbred gilts at a relative abundance of 2.0%^8^ and was also noted in non-pregnant sows^6^. Further data is needed to confirm that changes observed are associated with differences in PS.

Differences between this study and that performed in non-pregnant females are congruent with observations of shifts in microbial populations during pregnancy and parturition in humans^18^. An increase in *Treponema* OTUs in the PS3 sows was noted in this study though it is unknown if the higher abundance of *Treponema* OTUs in PS3 sows is associated with a higher risk for POP. It is noteworthy to mention that in humans *Treponema pallidum* is the causative agent of syphilis^19^. Furthermore, an increased abundance of *Treponema* on the vaginal microbiota has previously been associated with genital disease and reproductive disorders in cattle^20^. Interestingly, *Treponema* has been linked to swine dysentery^21^, a severe infectious disease that is often characterized by inflammation in the large intestine. While functional association with POP needs to be verified in future studies, the results presented herein highlight candidate phylotypes for future attention.

Interaction between the microbiome and host has become an increasing area of interest. The vaginal microbiome, in particular, is being investigated in humans in relation to reproductive disease, and this work has begun to transfer into livestock. Further, compromised human reproductive health, including pelvic inflammatory disease (PID), has been linked to changes in the vaginal microbiome^10^ and a biological link between pelvic infections and a disequilibrium of the vaginal flora is thought to exist^22^. However, PID is not the only disease linked to a disruption of the vaginal microbiome. Bacterial vaginosis (BV) in humans, although resulting from different pathogenic infections, has also been linked to altered vaginal microbiome^23,24^. It has been reported that microorganisms related to BV have been linked to an elevated risk of acquiring PID^25^. Vaginal microbiota consistent with BV or other changes in the vaginal microbiome are associated with an increased risk for viral sexually transmitted diseases and subsequent ascending infections^10,23^. As a result, BV contributes to endometritis and cervical inflammation which includes immune cell infiltration and localized erythema. Additionally, sexually transmitted infections have been linked to alterations in the vaginal microbiota^23,26^. Humans suffering from BV are reported to have substantial increases in *Prevotella*^27^, which was also elevated in PS3 sows. Differences in relative abundance of *Prevotella* and *Fusobacterium* in the vaginal microbiome between PS1 and PS3 sows was also discovered. Both of these microbial genera have been reported to be associated with infections caused by gram negative bacteria^28^, relevant since these are associated with BV and PID. Identifying differences in the vaginal microbiome associated with phenotypes of differential POP risk creates a starting point to mechanistically explore the contribution of vaginal dysbiosis to POP in swine.

In addition to characterization of the vaginal microbiome in sows with variable risk for POP, changes in components in circulation may also provide understanding of physiological contributions to POP risk. Serum contains a plethora of information about various small molecules suggesting the serum metabolome may have value as a predictive phenotype, particularly if coupled with microbial changes. Changes observed in the metabolome of a diseased individual may serve as primary indicators and in some cases are already used in clinical practice^29^. Alterations to the serum metabolome between sows with high and low risk for POP were observed in this study, known and potentially novel unidentified POP risk associated molecules. Similar to observations of the vaginal microbiome, major shifts in the overall serum metabolome were not observed, which was anticipated as only a subset of metabolites would be expected to be associated with such a specific phenotype in otherwise consistent biological samples. In this study, 26 metabolites were discovered which differed between low and high POP risk animals, 21 of which were increased in high risk of POP sows.

Small molecules within circulation may be useful to help differentiate between diseased and non-diseased status^30^. Circulating glucose levels have been reported to change during BV^27^. In our study of the serum metabolome, changes in glucose and its derivatives were identified between PS1 and PS3 animals. Vulvo-vaginal candidiasis (VVC) in humans has similar effects on the metabolome as BV^27^. Changes in lactic acid as well as changes in amino acids were determined in this study, suggesting molecular feature differences could be valuable in assessing reproductive dysfunction in sows. Increased abundance of 2-aminobutanoic acid, a derivative of butyric acid, which has been shown to correlate with inflammation^31,32^, was noted in PS3 sows compared to PS1 sows. Changes in the serum metabolome associated with reproductive dysfunction along with changes in the vaginal microbiome have been reported^18,27^ consistent with the findings reported herein.

Collectively, this study established a phenotypic scoring system to identify sows with differential POP risk. This approach enabled identification of biological contributors to POP risk which could serve as reliable POP biomarkers. Development of the scoring system will have utility for both producers and researchers. In addition, several bacterial and metabolomic candidates of interest have been discovered and the vaginal microbiome of pregnant sows has been established. The putative markers identified in this work will require determination of causality. Further, a clear biological relationship between the vaginal microbiome and serum metabolite differences is a future research avenue and addition of POP risk adds a further layer of both complexity and translatability.

## Materials and methods

### Animals

All experiments in this study were approved by the Iowa State University Institutional Animal Care and Use Committee and all methods were conducted in accordance with relevant guidelines and regulations. Two hundred and thirteen pregnant females (gestation days 105–117) from two commercial sow farms were used. All animals were individually housed in the commercial sow farms.

### Perineal scoring system

**Figure 7:**
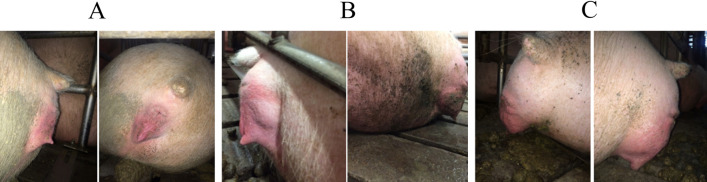
Perineal scoring (PS) methodology as an indicator of pelvic organ prolapse (POP). The perineal region was visually evaluated for swelling, redness and protrusion to assign scores. (**A**) If a sow lacked swelling, redness and protrusion they were considered low risk for POP and assigned as PS1. (**B**) Sows with some characteristics such as moderate swelling, redness and protrusion of the perineal area were considered moderate risk for POP and assigned as PS2. (**C**) Sows demonstrating all of the characteristics of severe swelling, redness and protrusion of the perineal area were assigned to the PS3 category and considered high risk for POP.

To categorize sows with differing risk of POP, a perineal scoring system was developed to assign sows into low (PS1), moderate (PS2) or high (PS3) POP risk (Fig. 7). To assign the score, the perineal region was visually evaluated for swelling, redness and protrusion. If a sow lacked swelling, redness and protrusion they were considered low risk for POP and assigned as PS1. Sows with some characteristics such as moderate swelling, redness and protrusion of the perineal area were considered moderate risk for POP and assigned as PS2. Sows demonstrating all of the characteristics of severe swelling, redness and protrusion of the perineal area were assigned to the PS3 category and considered high risk for POP. Importantly, a sow scoring a PS3 would be considered abnormal for any stage of gestation (Fig. 7). To minimize variation, animals were scored at one time point during the farm visit by the same individual, while the sow was lying down, and performed on animals between days 105 and 117 of gestation. The variation in gestation stage evaluation was the result of the timing of the visit to the farm and the emphasis was placed on sows within two weeks of expected farrowing date. Subsequently, the average parity was 3.04 ± 0.21 and the average gestation day was 110.7 ± 0.5, neither of which were significant between PS (*P* > 0.3). Animals chosen for sampling were parity matched. Animals were then monitored for subsequent POP occurrence during and following parturition.

### Sample collection

Vaginal swabs for microbiota DNA extraction and blood for serum collection were collected from sows classified as PS1 (n = 29) or PS3 (n = 23). Vaginal swabs were collected by aseptically inserting a 7-inch histology brush (2199, Puritan Medical Products) into the vagina and brushing the vaginal orifice for approximately 15 s. Swabs were removed and immediately placed in sterile 1 × phosphate buffered saline (PBS), frozen on dry ice, and stored at − 80 °C until pelleted and used for DNA extraction. Blood samples were collected by jugular venipuncture into vacutainer tubes, which were kept at room temperature to allow appropriate clotting before separation of serum. All serum samples were processed by centrifugation at 20 °C for 15 min at 2000×*g* followed by aspiration of serum for storage at − 80 °C until further analysis.

### DNA extraction

Tubes containing vaginal microbiota swabs in PBS were thawed, vortexed for 5 min to detach cells, and centrifuged at 5000×*g* at room temperature for 15 min to form a pellet. Supernatant was discarded and pellets were resuspended in 1 mL of sterile PBS and DNA was extracted with a Qiagen DNeasy Powerlyzer Powersoil kit (Qiagen, Germantown, MD) per manufacturer’s protocol. Briefly, mechanical cell lysis was performed using a Fisher Scientific Beadmill 24. The concentration of the isolated DNA was measured using a Qubit 3.0 Fluorometer and diluted to 25 ng/µL for each sample.

### 16S rRNA gene sequencing

16S rRNA gene sequences were amplified from the vaginal swab samples of individual animals. PCR amplification of the V4 region of bacterial 16S rRNA genes was performed with the conserved primers [515F (5′-GTGYCAGCMGCCGCGGTAA-3′^33^) and (806R (5′-GGACTACNVGGGTWTCTAAT-3′^34^) as previously described^35^] with sequence tags (bar codes) and sequencing primers incorporated into each PCR primer. PCR mixtures contained 200 µM (each) deoxyribonucleotide triphosphate, 2.0 µM (each) primer, 2.0 U Ampligold Taq polymerase (Applied Biosystems, Foster City, CA), 2.5 mM MgCl2, 50 ng template DNA, Ampligold Taq buffer (Applied Biosystems), and sterile water to 50 µl. PCRs were performed in a PTC-225 thermal cycler (BioRad DNA Engine Dyad Peltier Thermal Cycler) with the following protocol: 3 min at 95 °C; 21 cycles of 1 min at 95 °C, 30 s at 56 °C, and 45 s at 72 °C; and a final elongation step for 3 min at 72 °C. All PCR products were purified with a QIAquick 96 PCR Purification Kit (Qiagen, Hilden, Germany) according to the manufacturer’s instructions. PCR bar-coded amplicons were mixed at equal molar ratios and used for Illumina MiSeq paired-end sequencing with 250 bp read length and cluster generation with 10% PhiX control DNA on an Illumina MiSeq platform.

### Sequence analysis

Sequence analysis was performed with Mothur V1.40.4 following the Mothur MiSeq Standard Operating Procedure^35^. Barcode sequences, primers and low-quality sequences were trimmed using a minimum average quality score of 35, with a sliding window size of 50 bp. Chimeric sequences were removed with the “Chimera.uchime” command. For alignment and taxonomic classification of OTUs, the SILVA SSU NR reference database (V132) provided by the Mothur website was used. Sequences were clustered into OTUs with a cutoff of 99% 16S rRNA gene similarity (= 0.01 distance) and were ordered from most to least abundant. Representative sequences for the 50 most abundant OTUs were classified using NCBI BLAST, to improve classification accuracy.

### Gas chromatography mass spectrometry (GC–MS)

Serum samples from the identical sows used for microbiota analysis were analyzed by non-targeted GC–MS at the Iowa StateUniversity W.M. Keck Metabolomics Research Laboratory. Sample preparation was conducted using a modified version of the methanolic extraction and sample preparation was conducted using a modified version of the methanolic extraction and sample preparation methods established by Jiye et al.^36^. Serum (300 µL) from each sow was spiked with internal standards (10 µg of nonadecanoic acid and 10 µg ribitol (Sigma-Aldrich CO., St. Louis, MO)) and extracted twice using 0.8 mL cold methanol (Fisher Scientific, Waltham, MA), extracts were pooled and dried (0.8 mL of each extract) overnight using a SpeedVac system in preparation for gas chromatography with tandem mass spectrometry (GC–MS) analysis. The dried extracts were subjected to methoximation with methoxyamine hydrochloride (50 µL of 20 mg/mL in pyridine (Sigma-Aldrich CO., St. Louis, MO)) at 30 °C for 90 min. Samples were silylated with 70 µL BSTFA/1% TCMS (Sigma-Aldrich CO., St. Louis, MO) at 42 °C for 30 min, and then subjected to GC–MS analysis on an Agilent 7890C gas chromatograph in tandem with a 5975C MSD. The GC oven program began at 80 °C and was ramped at 5 °C/minute to 320 °C which was held for 6 min. The mass range was set from 40–800 m/z. The separation column was an HP5MSI (30 m long, 0.250 mm ID, 0.25 μm film thickness). The mass spectrometer operated under standard conditions with a 230 °C ion source. Identification and quantification were conducted using AMDIS with a manually curated retention indexed GC–MS library with additional identification performed using the NIST17 and Wiley 11 GC–MS spectral library.

### Statistical analyses

#### Effect of perineal score on subsequent pelvic organ prolapse

The Proc Mixed procedure in SAS was used to evaluate the effect of PS on the occurrence of POP by evaluating differences of least square means followed by comparisons between different PS using the probability of differences function.

#### Microbial sequence analysis

To compare alpha diversity between experimental groups, reads were randomly subsampled to accommodate the sample with the lowest number of reads across data sets (3000 sequences). Measurements of Chao species richness, Shannon Diversity, and Simpson evenness were taken to compare community structures between experimental groups. The means of the experimental group alpha diversity measures were compared using a pooled t-test assuming equal variance.

To compare overall microbial community structure, samples were given a Bray–Curtis dissimilarity value and means were then compared using the ANOSIM package provided by Mothur. Bray–Curtis was selected as the dissimilarity coefficient because of its ability to compare closely-related samples^37^.

All plotting was completed using ggplot2, v2_3.1.1 graphing package^38^ in R 3.6.0. Overall variation in bacterial communities was visualized using principle coordinate analysis (PCoA). Canonical correlation analysis (CCA) was used to visualize the variation strictly due to PS. This information was generated with the Phyloseq (v1.28.0^39^) and Vegan (v2.5–5^40^) packages using the shared and taxonomy file generated in Mothur. Bray–Curtis dissimilarity measures were used to generate distances between samples for the PCoA and CCA plots.

Differences in individual OTUs were compared using LEfSe^41^, identifying OTUs that most likely explain the greatest between-group variation. LEfSe performs a Kruskall-Wallis test to analyze all OTUs, broadly selecting OTUs that show the most variation between sample types. The retained features then undergo a pairwise Wilcoxon test, removing any OTUs that do not differ in ranking. In the last step, a linear discriminant analysis model is built from the retained OTUs to determine the effect sizes for each feature. P-values of < 0.05 were considered significant.

### Metabolite comparison analysis

MetaboAnalyst^42^, an R based statistical package was utilized to provide statistical evaluation of the GC–MS metabolite data. Important features were visualized using MetaboAnalyist’s univariate and multivariate analysis methods. For two-group data, fold change (FC) analysis, t-tests, and volcano plots, which is a combination of the first two methods, were produced. Hierarchical Clustering was created using the hclust function in package stat, and was displayed as a heatmap. Distances were calculated using Euclidean measures and clustering using the ward.D algorithm.

## Supplementary Information

Supplementary Information.

## Data Availability

The 16S rRNA gene sequences have been submitted to the NCBI Sequence Read Archive SRA and are available under the BioProject ID PRJNA623913.
