# Supplementary material for: Vaginal microbiome and serum metabolite differences in late gestation commercial sows at risk for pelvic organ prolapse
Source: Sci Rep. 2021 Mar 17;11:6189. doi: 10.1038/s41598-021-85367-3 (PMC7969946; doi:10.1038/s41598-021-85367-3)
Supplement: Supplementary file 1 — Supplementary Information. [file 41598_2021_85367_MOESM1_ESM.pdf]

Vaginal microbiome and serum metabolite differences in late gestation commercial sows at risk for pelvic organ prolapse

Supplementary Tables and Figures

Authors

Zoë E. Kiefer[1], Lucas R. Koester[2,3], Lucas Showman[4], Jamie M. Studer[1], Amanda L. Chipman[5], Aileen F. Keating[1], Stephan Schmitz-Esser [1,3], Jason W. Ross*,[1,5]

[1] Department of Animal Science, Iowa State University, Ames, Iowa, United States
[2] Department of Veterinary Microbiology and Preventive Medicine, Iowa State University
[3] Interdepartmental Microbiology Graduate Program, Iowa State University
[4] W. M. Keck Metabolomics Research Laboratory, Iowa State University
[5] Iowa Pork Industry Center, Ames, Iowa

Grant Support: This project was supported in part by the National Pork Board and Foundation for Food and Agriculture Research.

Correspondence:
Jason W. Ross
Department of Animal Science, 2356 Kildee Hall, Iowa State University, Ames, IA 50011, USA
Phone: 515-294-8647
Fax: 515-294-4471
Email: jwross@iastate.edu

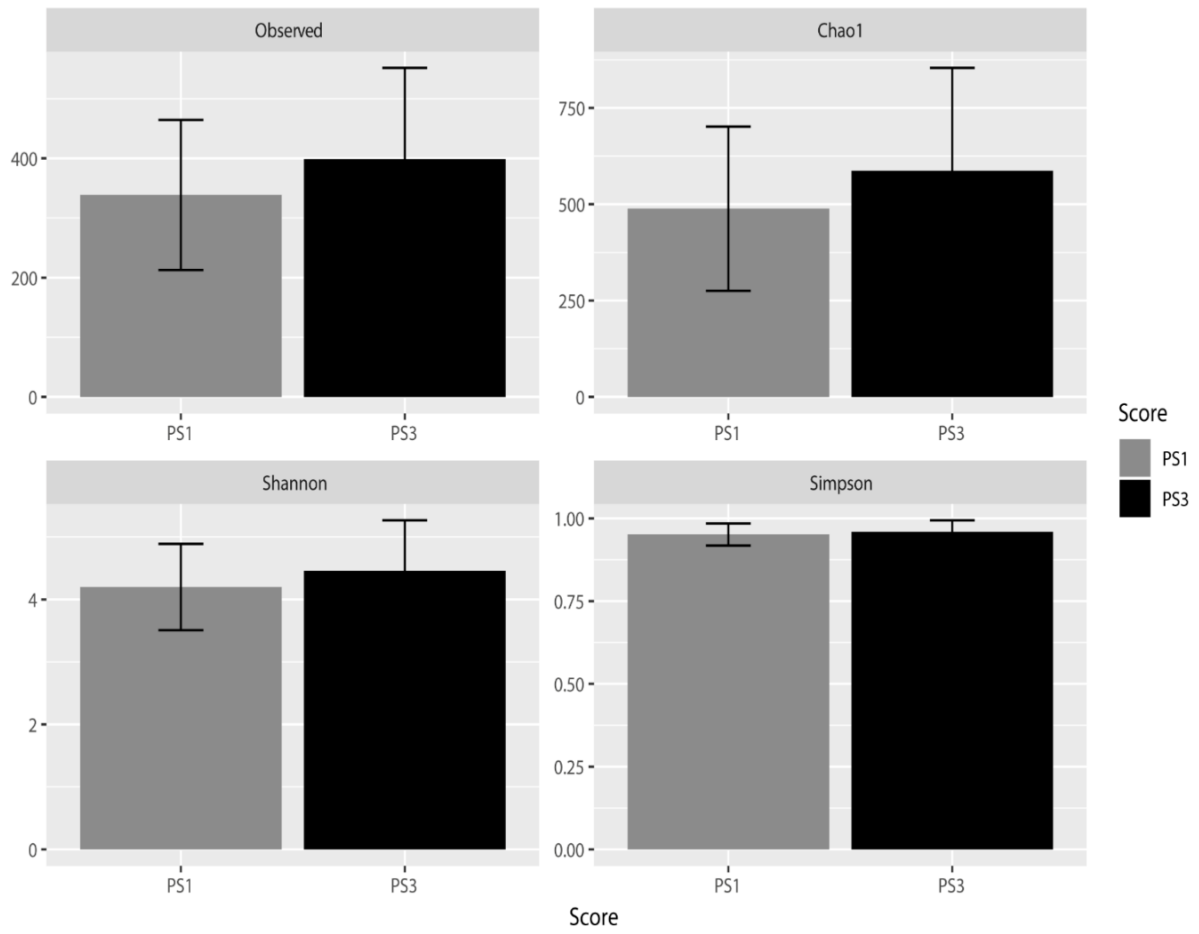

Supplementary Figure 1. Alpha diversity of vaginal microbiome for sows with low and high risk for prolapse. The samples show species richness and diversity. Chao species richness is an estimate of the true species richness based on the sample size and Shannon and Simpson analysis evaluate diversity.

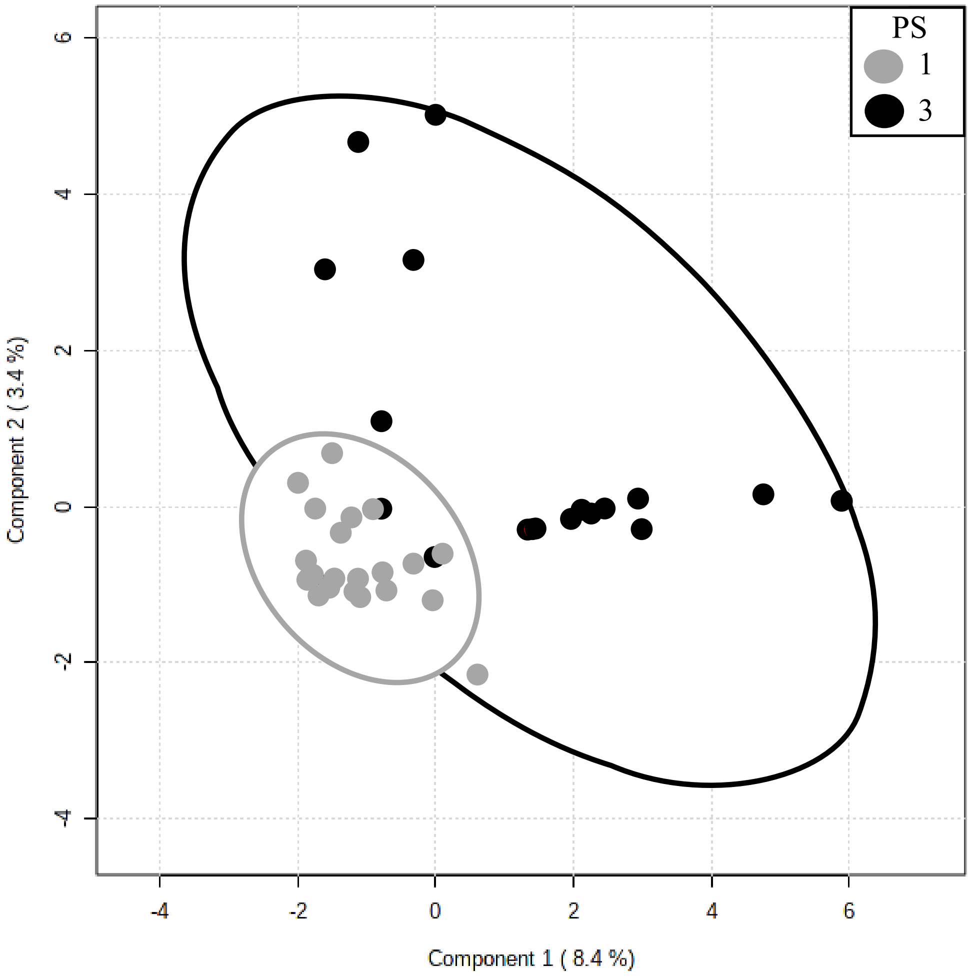

Supplementary Figure 2. Serum metabolite differences overview. Sparse Partial Least Squares Discriminant Analysis (SPLS-DA) was produced by the MetaboAnalyst program using the swine prolapse serum data. Grey dots represents sows with low risk for POP (perineal score 1 (PS1)), and animals with high risk (PS3) for POP are represented by the black dots. This figure compares serum metabolites between PS1 and PS3 on an individual animal basis. These data suggest there is some overlap between the metabolites within PS, which is to be expected, but there are also differences. This figure was made using the following Xia, J., Wishart, D. Web-based inference of biological patterns, functions and pathways from metabolomic data using MetaboAnalyst. *Nat Protoc* **6,** 743–760 (2011). https://doi.org/10.1038/nprot.2011.319

Supplementary Table 1. Fifty most abundant OTUs[1] within swine late gestation vaginal communities.

OTU	Relative abundance (%)	Phylum	Taxonomy (Silva v132)	NCBI BLAST		
				Classification	Similarity (%)	Accession no.
OTU 1	6.19	*Firmicutes*	*Veillonella*	*Veillonella caviae* PV1	99.6	NR_025762.1
OTU 2	5.23	*Proteobacteria*	*Pasteurellaceae_unclassified*	*Actinobacillus porcinus* 35NTS	100	FJ437063.1
OTU 3	2.61	*Fusobacteria*	*Fusobacterium*	*Fusobacterium gastrosuis CDW1*	100	NR_146837.2
OTU 4	2.44	*Bacteroidetes*	*Prevotellaceae_UCG-001*	*Duncaniella sp. TLL-A3*	84.7	MK521456.1
OTU 5	2.18	*Firmicutes*	*Phascolarctobacterium*	*Phascolarctobacterium succinatutens YIT*	99.6	NR_112902.1
OTU 6	2.10	*Firmicutes*	*Clostridium_sensu_stricto_1*	*Clostridium sp. CL-2*	100	KF528156.1
OTU 7	2.02	*Firmicutes*	*Parvimonas*	*Parvimonas sp. KA00067*	99.6	KP192297.1
OTU 8	1.97	*Firmicutes*	*Turicibacter*	*Turicibacter sp. H121*	100	CP013476.1
OTU 9	1.72	*Bacteroidetes*	*Bacteroides*	*Bacteroides massiliensis DCW_SL_46*	96.1	MK424043.1
OTU 10	1.54	*Proteobacteria*	*Pasteurellaceae_unclassified*	*Pasteurella mairii strain CCUG 27189*	100	NR_042886.1
OTU 11	1.38	*Proteobacteria*	*Escherichia-Shigella*	*Escherichia coli*	99.2	AJ567617.1
OTU 12	1.23	*Firmicutes*	*Romboutsia*	*Romboutsia sp. ilealis*	100	LN555523.1
OTU 13	1.12	*Euryarchaeota*	*Methanobrevibacter*	*Methanobrevibacter sp. N58C*	100	LN610763.1
OTU 14	1.06	*Bacteroidetes*	*Bacteroidales_unclassified*	*Muribaculum sp. S4*	85.0	MK287698.1
OTU 15	1.03	*Firmicutes*	*Terrisporobacter*	*Bacterium WH2-11*	100	JQ269302.1
OTU 16	0.97	*Proteobacteria*	*Pasteurellaceae_unclassified*	*Pasteurella aerogenes ZWR1*	99.6	MN006266.1
OTU 17	0.96	*Bacteroidetes*	*Prevotellaceae_unclassified*	*Prevotella timonensis SSA5*	93.7	MH714863.1
OTU 18	0.81	*Firmicutes*	*Clostridium_sensu_stricto_1*	*Clostridium moniliforme HYN0057*	99.6	KY079341.1
OTU 19	0.79	*Firmicutes*	*Peptostreptococcus*	*Peptostreptococcus sp. DSM 106284*	99.6	MN537513.1
OTU 20	0.77	*Firmicutes*	*Lactobacillus*	*Lactobacillus sp. strain DSM 107288*	100	MN537532.1
OTU 21	0.72	*Firmicutes*	*Kurthia*	*Kurthia gibsonii EMB4*	100	KY048434.1
OTU 22	0.72	*Bacteroidetes*	*Porphyromonas*	*Porphyromonas somerae KA00683*	99.6	KP192301.1
OTU 23	0.70	*Bacteroidetes*	*Prevotellaceae_NK3B31_group*	*Prevotellaceae bacterium*	96.1	LC333724.1
OTU 24	0.69	*Actinobacteria*	*Corynebacterium_1*	*Corynebacterium xerosis FDAARGOS_674*	100	CP046322.1
OTU 25	0.66	*Firmicutes*	*Streptococcus*	*Streptococcus suis SS248*	100	KR819490.1

OTU 26	0.66	*Firmicutes*	*Jeotgalibaca*	*Jeotgalibaca dankookensis HM*	99.6	KU714711.1
OTU 27	0.63	*Bacteroidetes*	*Prevotella_1*	*Prevotellaceae bacterium strain AGP2-01-07-05*	99.2	MH699323.1
OTU 28	0.63	*Bacteroidetes*	*Prevotellaceae_NK3B31_group*	*Prevotellaceae bacterium*	98.4	LC333719.1
OTU 29	0.62	*Fusobacteria*	*Fusobacterium*	*Fusobacterium necrophorum FDAARGOS_565*	100	CP033837.1
OTU 30	0.60	*Firmicutes*	*Clostridiales_unclassified*	Unidentified *Eubacterium*	91.7	AJ229202.1
OTU 31	0.57	*Firmicutes*	*Anaerococcus*	*Anaerococcus tetradius*	99.6	LC036320.1
OTU 32	0.55	*Bacteroidetes*	*Prevotellaceae_unclassified*	*Prevotella sp. NAIP4D*	96.1	JQ797590.1
OTU 33	0.55	*Bacteroidetes*	*p-251-o5_ge*	*Parabacteroides distasonis WYJ14_D4*	85.2	MN081648.1
OTU 34	0.54	*Firmicutes*	*Clostridium_sensu_stricto_1*	*Clostridium butyricum CFSA3989*	100	CP033249.1
OTU 35	0.53	*Firmicutes*	*Clostridium_sensu_stricto_1*	*Clostridium celatum*	99.6	AB971795.1
OTU 36	0.52	*Proteobacteria*	*Acinetobacter*	*Acinetobacter defluvii WCHA30*	99.2	CP029397.2
OTU 37	0.49	*Firmicutes*	*Streptococcus*	*Streptococcus hyovaginalis TRG26*	100	MH329638.1
OTU 38	0.48	*Spirochaetes*	*Treponema_2*	*Treponema bryantii,*	99.6	AB849328.1
OTU 39	0.46	*Bacteroidetes*	*Bacteroides*	*Prevotella sp. cp02.11*	95.3	AY827858.1
OTU 40	0.45	*Firmicutes*	*Phascolarctobacterium*	*Phascolarctobacterium succinatutens*	99.2	AB490812.1
OTU 41	0.45	*Bacteroidetes*	*Prevotellaceae_NK3B31_group*	*Prevotellaceae bacterium*	98.8	LC333719.1
OTU 42	0.44	*Proteobacteria*	*Acinetobacter*	*Acinetobacter sp. MSRC7*	100	MH447439.2
OTU 43	0.43	*Firmicutes*	*Ruminococcus_1*	*Bacterium MA2007*	99.6	KF698129.1
OTU 44	0.43	*Bacteroidetes*	*Porphyromonas*	*Porphyromonas endodontalis*	96.4	LT680662.1
OTU 45	0.43	*Firmicutes*	*Kurthia*	*Kurthia gibsonii SAU_AFB01*	99.6	MN658386.1
OTU 46	0.42	*Bacteroidetes*	*Prevotellaceae_UCG-001*	*Muribaculum sp. S4*	86.1	MK287698.1
OTU 47	0.42	*Bacteroidetes*	*Bacteroides*	*Bacteroides fragilis LPB0329*	100	MN629228.1
OTU 48	0.42	*Bacteroidetes*	*Prevotella_9*	*Prevotellaceae bacterium AGP1-12-14-09*	100	MH699319.1
OTU 49	0.41	*Bacteroidetes*	*Porphyromonas*	*Porphyromonas katsikii JF5581*	91.3	KM360064.1
OTU 50	0.41	*Firmicutes*	*Terrisporobacter*	*Terrisporobacter sp. CCK3R4-PYG-107*	99.2	KR364793.1

[1]Individual microbes were assigned in order of abundance and classified into operational taxonomic units (OTUs)

Supplementary Table 2. Small molecule metabolites in serum from sows with differing risk of POP.

	Perineal Score[1]			
**Metabolite[2]**	**PS1**	**PS3**	**Fold Change**	***P*-value**
RI=1242.7, 11.7229 min, 35889	1.47E-13	7.78E-12	5.72	< 0.01
RI=1693.9, 17.2652 min, 20024	8.99E-13	3.54E-11	5.30	< 0.01
D-Fructose	1.48E-11	5.80E-10	5.29	< 0.01
RI=2105.9, 20.8495 min, 17937	1.04E-13	3.21E-11	8.27	< 0.01
RI=1208.7, 11.2167 min, 40555	4.75E-14	3.51E-12	6.21	< 0.01
RI=1915.0, 19.2855 min, 17937	2.26E-12	1.31E-10	5.86	< 0.01
RI=1180.6, 10.7977 min, 36473	2.04E-12	7.66E-11	5.23	< 0.01
RI=2109.7, 20.8792 min, 17937	1.49E-13	3.33E-11	7.81	< 0.01
RI=1758.2, 17.8880 min, 40555	4.25E-14	3.85E-12	6.50	< 0.01

[1]Sows were assigned a perineal score based on their relative risk of experiencing a pelvic organ prolapse (POP). Sows assigned PS1 were presumed low risk for POP while sows assigned PS3 were presumed high risk for POP.

[2]Metabolite list only includes those that were identifiable and statistically significant ($P < 0.01$) between perineal scores. Values reported as moles per mg.
